# Ratiometric and colorimetric near-infrared sensors for multi-channel detection of cyanide ion and their application to measure β-glucosidase

**DOI:** 10.1038/srep16528

**Published:** 2015-11-09

**Authors:** Panfei Xing, Yongqian Xu, Hongjuan Li, Shuhui Liu, Aiping Lu, Shiguo Sun

**Affiliations:** 1 College of Science, Northwest A&F University, Yangling, Shaanxi, 712100, China; 2 School of Chinese Medicine, Hong Kong Baptist University, Kowloon Tong, Hong Kong, 999077, China

## Abstract

A near-infrared sensor for cyanide ion (CN^−^) was developed via internal charge transfer (ICT). This sensor can selectively detect CN^−^ either through dual-ratiometric fluorescence (logarithm of I_414_/I_564_ and I_803_/I_564_) or under various absorption (356 and 440 nm) and emission (414, 564 and 803 nm) channels. Especially, the proposed method can be employed to measure β-glucosidase by detecting CN^−^ traces in commercial amygdalin samples.

Cyanide is an important raw material of pharmaceuticals, insecticides and fertilisers, as well as an indispensable reagent in metallurgy and gilded industry^1^,^2^. However, CN^−^ can bind into mitochondrial cytochrome oxidase and inhibit critical enzymatic cascades in organisms, thus, CN^−^ is extremely detrimental. Consequent intracellular hypoxia often leads to central nervous system disorders, cardiovascular impairment or even death by suffocation within seconds to minutes^3^. It should be noted that CN^−^ can be derived from a variety of sources such as those from industrial accidents, pharmaceuticals, fire smoke and even some common foods^4^. For example, ingested amygdalin, which is one of the most typical cyanogenic glycosides found in kernels such as peach, apricot, cherry and almond, would be hydrolysed by the widely distributed natural β-glucosidase and generated hydrogen cyanide (HCN) in living organisms, leading to disruption of normal physiological activities and may result in death caused by rapid suffocation^5^. Therefore, CN^−^ monitoring has attracted much attention, and many techniques such as voltammetry, titrimetry, electrochemical and chromatography have been developed^6^,^7^,^8^,^9^,^10^. Unfortunately, most of these methods require expensive equipment and are complicated for real-time analyses.

Recently, fluorometric detection has been increasingly used because of its low cost and rapid operation. In 2005, Geddes *et al.* reported a set of sensors for CN^−^ detection based on complexation between boronic acid and CN^−^11. In 2008, Qin *et al.* described a reversible probe for CN^−^ based on the conversion of zinc ions in the presence of Cu^2+^ and CN^−^12. On the basis of nucleophilic attack of the hydrogen-bonded carbonyl groups, Lee *et al.* developed a new method to detect CN^−^ in 2009^13^. In 2011, Lee *et al.* selectively detected CN^−^ based on the addition reaction between indole moiety and CN^−^14. With a similar mechanism, Shiraishi *et al.* recently synthesised a new fluorescent probe to selectively and sensitively detect CN^−^15. Most of these probes are designed on ON-OFF and OFF-ON mechanisms^16^,^17^,^18^,^19^,^20^,^21^, wherein detection depends only on the changes in single emission and might be affected by external factors including excitation power, detector sensitivity and environment^22^.

According to literature^14^,^15^,^23^, CN^−^ can conjugate with fluorescent dyes to modulate the ICT state and also elicit selective fluorescence changes. Thus, a novel fluorometric sensor **SY** (Fig. 1) was designed. **SY** displays an instantaneous (<5 s) fluorescence signal change in the presence of CN^−^ (0–10 μM) in acetonitrile, and a unique dual-ratiometric fluorescence (I_414_/I_564_ and I_803_/I_564_) can be achieved for more accurate detection, especially that CN^−^ can be measured through multi-channels (under two absorption wavelengths of 356, 440 nm and three emission wavelengths of 414, 564, 803 nm), offering versatility during measurement. The applicability of **SY** was verified by its successful application in measuring β-glucosidase based on the above-mentioned typical hydrolysing ability towards amygdalin, which is useful in monitoring β-glucosidase level in living organisms because abnormal changes in β-glucosidase are linked to Gaucher’s disease, Parkinson disease, pulmonary hypertension and even central nervous system disorders^24^,^25^.

## Results

### UV-vis absorption titration spectra of SY upon addition of CN^−^

**Figure 2:**
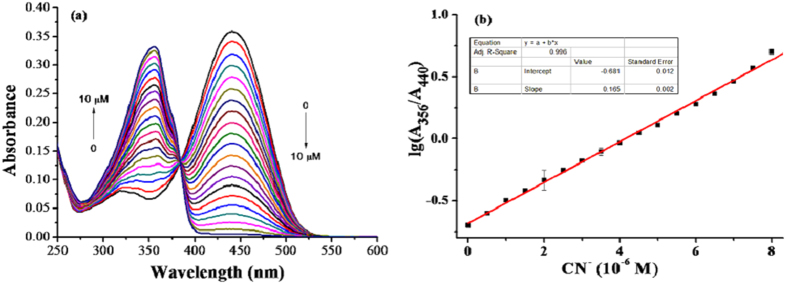
(**a**) UV-vis absorption titration spectra of **SY** (5 μM) in the presence of CN^−^ (0–10 μM) in acetonitrile at room temperature; (**b**) The linear relationship between the logarithm of the absorbance values (A_356_/A_440_) and CN^−^ concentration (0–10 μM).

Firstly, the reaction time between **SY** and CN^−^ was tested. As shown in Fig. S1 (ESI†), **SY** can complete the reaction with CN^−^ within 5 s, indicating a rapid response of **SY** to CN^−^, which is beneficial in real-time detection. The UV-vis absorption titration spectra of **SY** (5 μM) were measured in acetonitrile at room temperature. As shown in Fig. 2a, the absorbance at 440 nm gradually decreased with the addition of CN^−^ and a new peak at 356 nm appeared, indicating that a chemical reaction occurred between CN^−^ and the indole group of **SY**. A colour change from yellow to colourless was dependent on CN^−^ concentration (Fig. S6), and 10 μM CN^−^ led to saturation. The logarithm of the absorbance ratio (A_356_/A_440_) produced a linear relationship (R^2^ = 0.996) with CN^−^ concentration ranging from 0 μM to 7.5 μM (Fig. 2b), and the detection limit for CN^−^ based on 3σ/slope was 9.1 × 10^−8^ M^26^. In addition, the absorption values at each single peak also showed excellent linear relationship with R^2^ = 0.998 (356 nm, 0–10 μM CN^−^) and R^2^ = 0.996 (440 nm, 0–10 μM CN^−^) respectively (Fig. S2). The detection limit was 5.8 × 10^−7^ M at 356 nm and 3.9 × 10^−7^ M at 440 nm under similar condition.

### Fluorescence titration studies of **SY** upon addition of CN^−^

**Figure 3:**
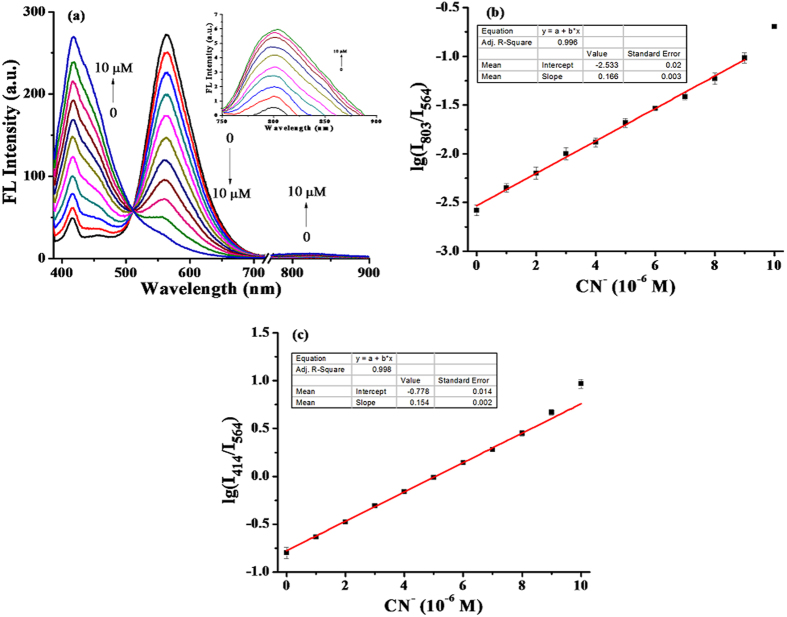
The fluorescence titration studies of SY towards CN^−^. (**a**) Fluorescence spectra of **SY** (5 μM) upon addition of CN^−^ (0–10 μM) at room temperature. Inset: enlarged fluorescence spectra from 750 nm to 900 nm, λ_ex_ = 360 nm. (**b**) The corresponding linear relationship between the logarithm of the fluorescence ratio (I_803_/I_564_) in the presence of CN^−^ (0–9 μM). (**c**) The corresponding linear relationship between the logarithm of the fluorescence ratio (I_414_/I_564_) in the presence of CN^−^ (0–10 μM).

Secondly, fluorescence titration studies on **SY** (5 μM) towards CN^−^ (0–10 μM) were also performed, and a trend similar to that in UV-vis absorption titration was observed. With the addition of CN^−^, the peak at 564 nm gradually decreased, and a new emission peak at 414 nm was recorded. It’s worth noting that a concentration-dependent emission at 803 nm gradually and evidently increased owing to the nucleophilic attack of CN^−^, which destroyed the conjugation of **SY** and blocked the ICT (Fig. 3a, Fig. S9–14)^27^. Accordingly, an excellent linear relationship (R^2^ = 0.996) was fitted between the logarithm of the fluorescence ratio (I_803_/I_564_) and CN^−^ concentration from 0 μM to 9 μM (Fig. 3b). The detection limit (3σ/slope) for CN^−^ was 7.3 × 10^−8^ M^28^. A second linear relationship (R^2^ = 0.998) was also obtained between the logarithm of the fluorescence ratio (I_414_/I_564_) and CN^−^ concentration ranging from 0 μM to 10 μM at a detection limit of 5.4 × 10^−8^ M (Fig. 3c). As shown in Fig. S3 and S4, **SY** exhibited a good corresponding linear relationship with CN^−^ under each of the following emission channels: R^2^ = 0.998 (414 nm, 0–10 μM CN^−^), 0.998 (564 nm, 0–10 μM CN^−^) and 0.995 (803 nm, 0–10 μM CN^−^). The **SY** performance was subsequently studied in a mixing solvent of acetonitrile and increasing amount of water (0–20%, V/V, Fig. S5). Overall, the results verified the stability of **SY**.

### Competitive experiments for CN^−^ selectivity

Furthermore, S^2−^, CO_3_^2−^, ACO^−^, S_2_O_3_^2−^, SO_4_^2−^, I^−^, Br^−^, Cl^−^, H_2_PO_4_^−^, F^−^, SCN^−^, NO_2_^−^, glutathione (GSH), homocysteine (Hcy) and cysteine (Cys) were selected as interference ions to evaluate the selectivity of **SY**^15^,^22^. As shown in Fig. S7, with the addition of these various analytes (25 μM for each), **SY** did not show any significant fluorescence changes at 414 and 803 nm. By contrast, the addition of only 5 μM CN^−^ enhenced fluorescence at 414 and 803 nm, indicating that **SY** can selectively detect CN^−^.

## Discussion

^1^H NMR and MS were used to verify the mechanism of **SY** in CN^−^ detection. As shown in Fig. S15a, the protons of the benzene ring in **SY** exhibited an upfield shift upon addition of CN^−^. Similar results were observed on the protons of methyl and methylene groups (Fig. S15b), indicating the function of CN^−^. The mass spectra (Fig. S24) further proved the **SY-CN** formation, *m/z* 462.36 (calc. = 462.20) corresponding to [**SY** + CN + H]^+^.

### Application for measuring enzyme activity *in vitro*

**Figure 4:**
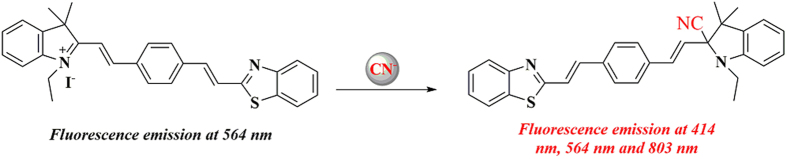
The mechanism of SY for CN^−^.

To confirm the applicability of the proposed method, **SY** was employed to study the activity of β-glucosidase by using cyanogenic glycoside amygdalin as indicator and **SY** as signal receptor^29^. Moreover, β-Glucosidase catalysed the hydrolysis of amygdalin to free CN^−^, initiating a signal change in the fluorescence spectrometer resulting from the reaction between **SY** and CN^−^ (Fig. 4). As shown in Table 1, the amount of CN^−^ detected by **SY** (the logarithm of the fluorescence ratio I_414_/I_564_, I_803_/I_564_) matched the concentrations of β-glucosidase. The relative standard deviations (RSDs) of I_414_/I_564_ and I_803_/I_564_ were lower than 4.9% and indicative of fine accuracy, further demonstrating the potential application of this method in CN^−^ determination. An alternative method (I_414_, I_564_ and I_803_, Table S1) was also provided to quantify CN^−^. However, the RSDs of I_414_, I_564_ and I_803_ were larger than those of I_414_/I_564_ and I_803_/I_564_, indicating that enhanced precision can be achieved by ratiometric method.

In conclusion, a novel near-infrared probe **SY** for CN^−^ was successfully developed. **SY** exhibits a unique colorimetric in UV-vis and dual-ratiometric in fluorescence towards CN^−^, resulting in more accurate detection. Moreover, its multi-channel detection offers versatility during measurement. The minimum detection limit of **SY** was 5.4 × 10^−8^ M. Furthermore, **SY** was successfully used to measure β-glucosidase. Overall, the results indicate that **SY** is a selective sensor for CN^−^ and a potential indicator of β-glucosidase.

## Methods

### Synthesis material and instruments

All solvents and chemical reagents were analytical grade and purchased from commercial suppliers. ^1^H NMR and ^13^C NMR spectra were recorded on a Bruker 500 AVANCE III spectrometer with chemical shifts reported in ppm at room temperature. Mass spectra were obtained with Thermo Fisher LCQ Fleet mass spectrometer (USA) and a LC/Q-Tof MS spectrometry (USA). Absorption spectra were collected by using a Shimadzu 1750 UV-visible spectrometer (Japan). Fluorescence spectra were measured with a Shimadzu RF-5301 fluorescence spectrometer (Japan) with both excitation and emission slits set at 10 nm if without further demonstration.

Compound **(A)** and **(B)** is prepared according to the reported literature^30^,^31^. Synthesis of **(A)** A mixture of 2-methylbenzimidazole (1.33 g, 5 mmol), p-phthaldialdehyde (1.34 g, 5 mmol) were added into 1.5 mL acetic anhydride and 1 mL acetic acid, stirring at 120°C for 6 h. Cooled to room temperature, 5 mL concentrated hydrochloric acid was added. Then 30% aqueous sodium hydroxide solution to make pH = 7, filtered and washed with water. The crude product was purified by column chromatography on silica gel using DCM to obtain a yellowish green crystal, 65% yield. ^1^H NMR (500 MHz, DMSO-*d*_*6*_) δ 10.02 (s, 1 H), 8.11 (d, *J* = 6.6 Hz, 1 H), 8.00 (dd, *J* = 6.6, 2.3 Hz, 3 H), 7.94 (d, *J* = 6.6 Hz, 2 H), 7.78 (q, *J* = 13.2 Hz, 2 H), 7.58–7.49 (m, 1 H), 7.48–7.40 (m, 1 H).

Synthesis of **(B)** 2,3,3-trimethylindolenine (1 mL, 5.3 mmol) and iodoethane (0.9 mL, 10.6 mmol) were mixed in CH_3_CN (15 mL), refluxing for 24 h. Cooled to room temperature, filtered, washed with ethyl acetate (3 × 10 mL) to obtain a pink crystal, 85% yield.

Synthesis of **SY** A mixture of **(A)** (0.5 mmol), **(B)** (0.5 mmol) and anhydrous sodium acetate (1 mmol) were added into 3 mL acetic anhydride, stiring at room temperature for 10 h. And then filtered, washed with water. The crude product was purified by column chromatography on silica gel using CH_2_Cl_2_ and ethyl acetate (1:1, V/V) to obtain a red solid, 60% yield. ^1^H NMR (500 MHz, DMSO-*d*_*6*_) δ 8.47 (d, *J* = 13.0 Hz, 1 H), 8.32 (d, *J* = 6.5 Hz, 2 H), 8.13 (d, *J* = 6.5 Hz, 1 H), 8.01 (t, *J* = 6.5 Hz, 3 H), 7.96 (dd, *J* = 4.9, 2.3 Hz, 1 H), 7.93–7.86 (m, 2 H), 7.77 (dd, *J* = 13.0, 2.0 Hz, 2 H), 7.64 (dd, *J* = 4.9, 2.3 Hz, 2 H), 7.57–7.50 (m, 1 H), 7.49–7.43 (m, 1 H), 4.76 (q, *J* = 5.7 Hz, 2 H), 1.84 (s, 6 H), 1.50 (t, *J* = 5.7 Hz, 3 H). ^13^C NMR (125 MHz, DMSO-*d*_*6*_) δ 181.82, 166.49, 153.99, 153.30, 144.61, 140.91, 140.45, 136.55, 135.66, 134.83, 131.71, 130.11, 129.69, 128.86, 127.22, 126.29, 125.11, 123.66, 123.27, 122.83, 115.78, 113.37, 52.88, 42.89, 26.03, 14.38. HRMS: [M-I]^+^, calculated for [C_29_H_27_N_2_S], 435.1889, found: 435.1887.

## Additional Information

**How to cite this article**: Xing, P. *et al.* Ratiometric and colorimetric near-infrared sensors for multichannel detection of cyanide ion and their application to measure β-glucosidase. *Sci. Rep.* **5**, 16528; doi: 10.1038/srep16528 (2015).

## Supplementary Material

Supplementary Information

## Figures and Tables

**Figure 1:**
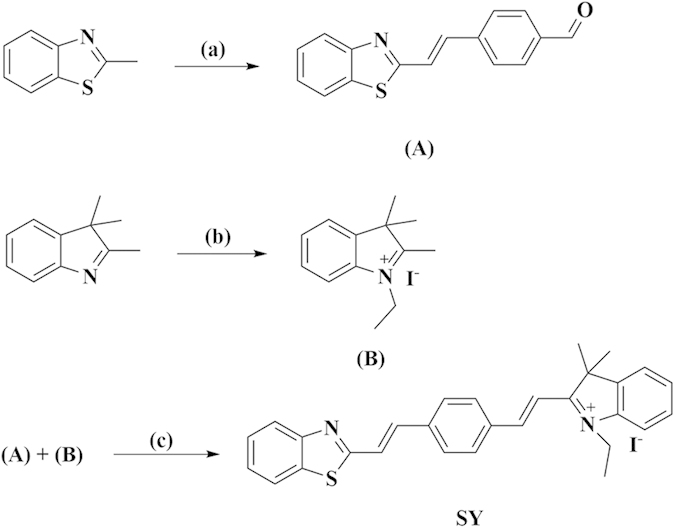
The synthesis of SY. Reagents and conditions: (a) 1,4-phthalaldehyde, acetic anhydride, acetic acid, 120°C, 6 h; (b) ethyl iodide, CH_3_CN, reflux, 24 h; (c) acetic anhydride, CH_2_Cl_2_, anhydrous sodium acetate, stirring, 10 h.

**Table 1:** β-Glucosidase assays in commercial amygdalin samples.

samples	β-glucosidase (mU)	theoretical value (μM)	CN^−^(I_414_/I_564_, μM)	RSD (I_414_/I_564_, %)	CN^−^(I_803_/I_564_, μM)	RSD (I_803_/I_564_, %)
			1.0		1.0	
1	20	1.0	1.0	4.9	1.1	4.9
			1.1		1.0	
			5.2		5.3	
2	100	5.0	5.1	3.3	5.2	2.7
			5.2		5.3	
			7.4		7.6	
3	150	7.5	7.2	1.3	7.4	1.7
			7.6		7.3	
