# Supplementary material for: Ratiometric and colorimetric near-infrared sensors for multi-channel detection of cyanide ion and their application to measure β-glucosidase
Source: Sci Rep. 2015 Nov 9;5:16528. doi: 10.1038/srep16528 (PMC4637889; doi:10.1038/srep16528)
Supplement: Supplementary Information [file srep16528-s1.pdf]

Electronic Supplementary Information (ESI)

Ratiometric and colorimetric near-infrared sensors for multi-channel detection of cyanide ion and their application to measure β-glucosidase

Panfei Xing,[a] Yongqian Xu,[a] Hongjuan Li,[a] Shuhui Liu,[a] Aiping Lu,[b*] and Shiguo Sun[a*]

[a] *College of Science, Northwest A&F University, Yangling, Shaanxi, 712100, China, E-mail: sunsg@nwsuaf.edu.cn*

[b]*School of Chinese Medicine, Hong Kong Baptist University, Kowloon Tong, Hong Kong, 999077, China, E-mail: aipinglu@hkbu.edu.hk*

Table of contents

1. **Sample preparation and titration**
2. **Synthesis of SOH**
3. **The calculation of LOD**
4. **Kinetics of fluoresence profile**
5. **^{1}H NMR, ^{13}C NMR and MS spectra**
6. **References**

1. Sample preparation and titration

Stock solutions of CN^- and other anions were prepared in deionized water. The concentration are fixed to 1.0 × 10^{-3} M. Stock solution of **SY** (1.0 × 10^{-3} M) were prepared in CH_3OH and then further diluted to 5.0 × 10^{-6} M for titration experiments. Every time an appropriate volume of each analyte was added to the test solution.

Hydrolysate was produced from 1 mL amygdalin aqueous solution (10 mM) upon addition different amount of β-glucosidase (final concentration 2.0 U/mL, 10.0 U/mL and 15.0 U/mL) and incubated at 37 ℃ for 50 min. Then 30 μL of each kind of hydrolysates was added into a solution of **SY** (5 μM) in acetonitrile (3 mL) for measurement. And every data was recorded after 5 min.

2. Synthesis of SOH

SOH was prepared following the reported procedure[1]. ^{1}H NMR (500 MHz, DMSO-d_6) δ 9.93 (s, 1H), 8.04 (d, *J* = 7.9 Hz, 1H), 7.93 (d, *J* = 8.0 Hz, 1H), 7.61 (d, *J* = 8.0 Hz, 2H), 7.55 (d, *J* = 16.0 Hz, 1H), 7.48 (t, *J* = 7.9 Hz, 1H), 7.38 (dd, *J* = 16.0, 8.4 Hz, 2H), 6.83 (d, *J* = 8.3 Hz, 2H).

3. The calculation of LOD

The detection limit was calculated based on the fluorescence titration according to the literature[2-4]. The fluorescence intensity of ten reagent blank samples containing no CN^- was measured, and the mean as well as the standard deviation (SD) was calculated.

$$LOD = 3\sigma/slope$$

Where σ is the standard deviation of the blank solution measured by 10 times; slope comes from the calibration curve.

4. Kinetics of fluoresence profile

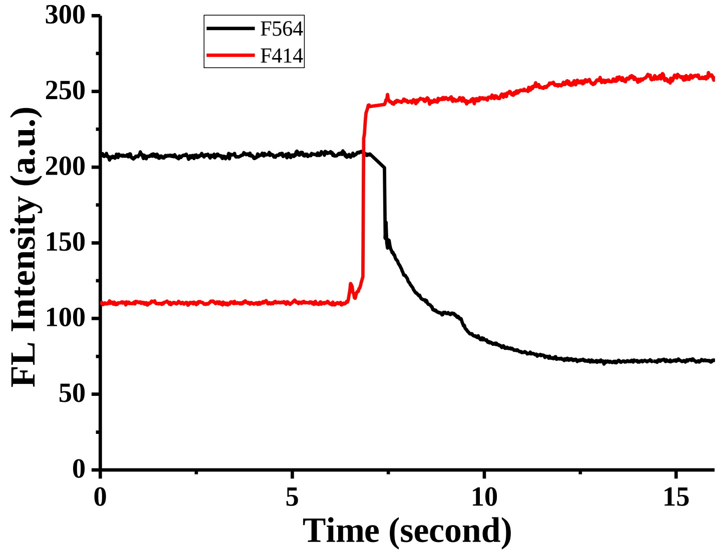

Fig. S1 The kinetic study of the fluorescence response of probe **SY** (5 μM) to CN^- (5 μM) in acetonitrile at 564 and 414 nm, λ_{ex} = 360 nm.

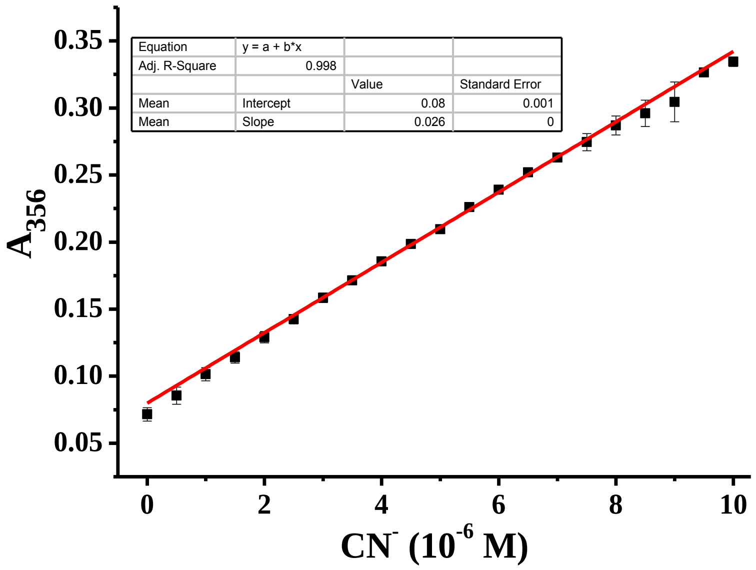

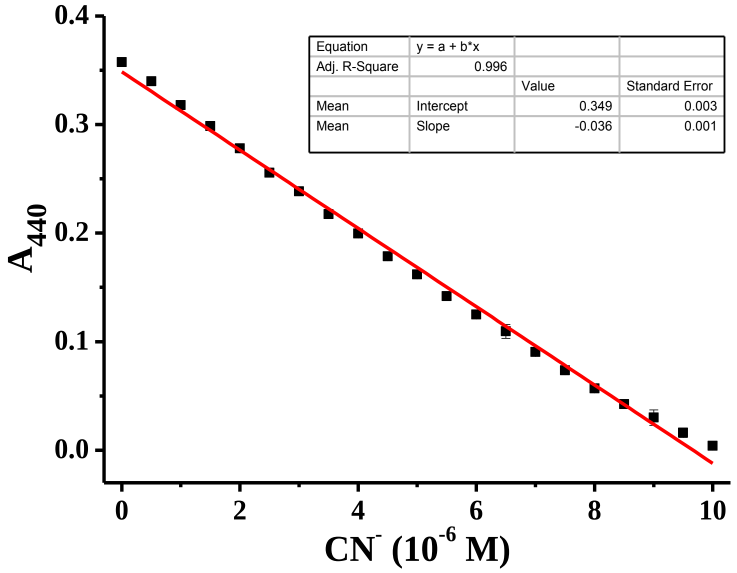

Fig. S2 The linear calibration curve between the absorbance value of **SY** (5 μM) at 356 nm (A_{356}, top figure), 440 nm (A_{440}, bottom figure) and the concentration of CN^{-} in the range of 0 μM to 10 μM.

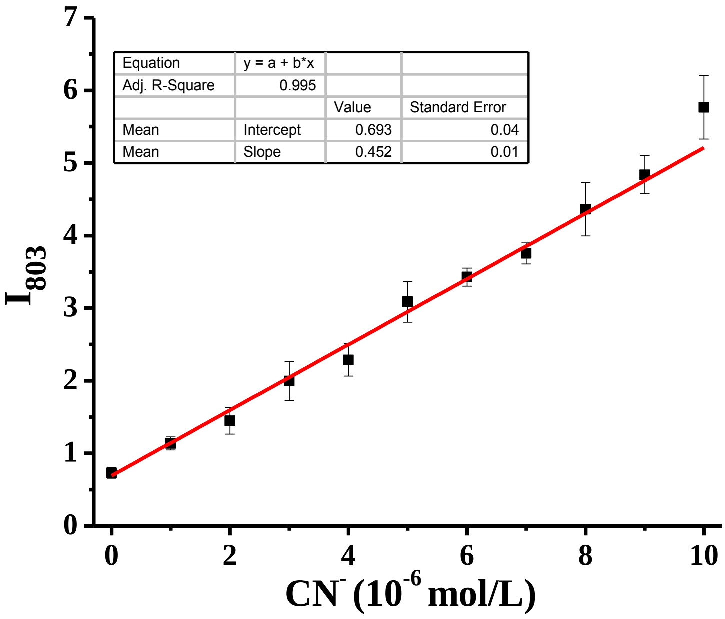

Fig. S3 The linear calibration curve between the fluorescence intensity of **SY** (5 μM) at 803 nm (I_{803}) and the concentration of CN^- in the range of 0 μM to 9.5 μM. λ_{ex} = 360 nm.

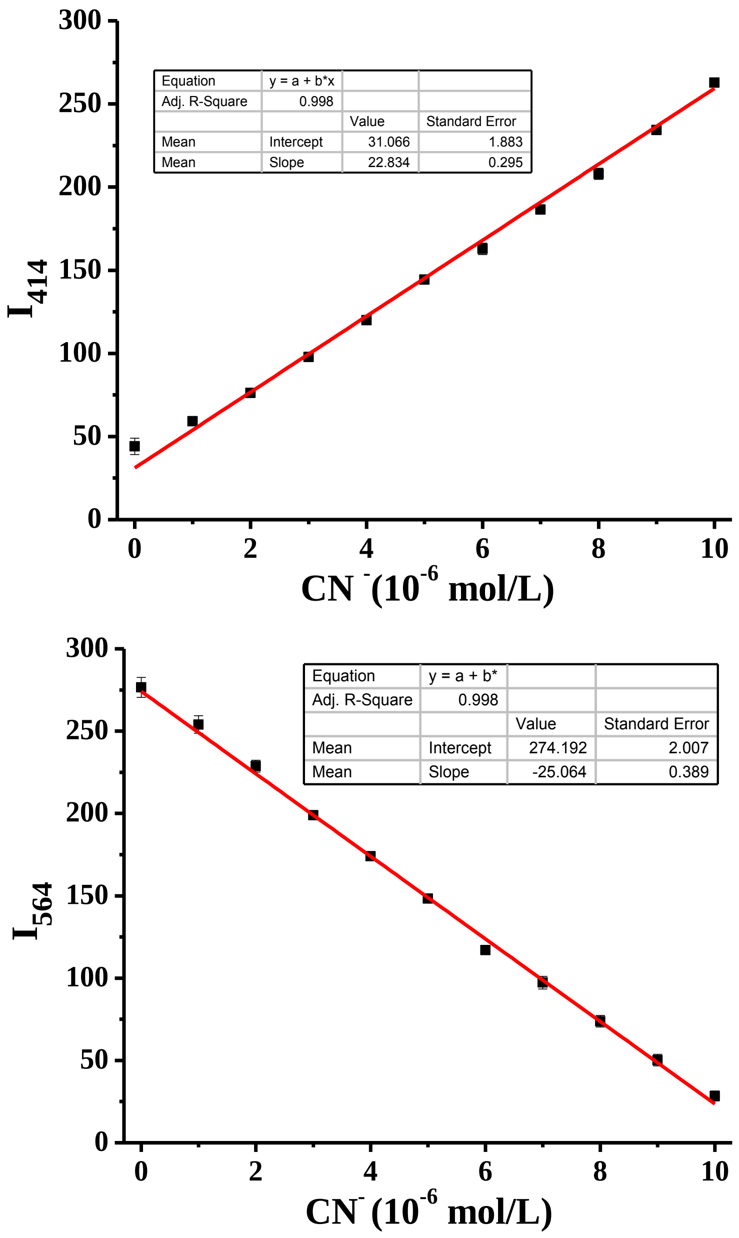

Fig. S4 The linear calibration curve between the fluorescence intensity of **SY** (5 μM) at 414 nm (I_{414}) and the concentration of CN^- in the range of 0 μM to 8.5 μM (top figure) and the linear calibration curve between the fluorescence intensity of **SY** (5 μM) at 564 nm (I_{564}) and the concentration of CN^- in the range of 0 μM to 6 μM (bottom figure). λ_{ex} = 360 nm.

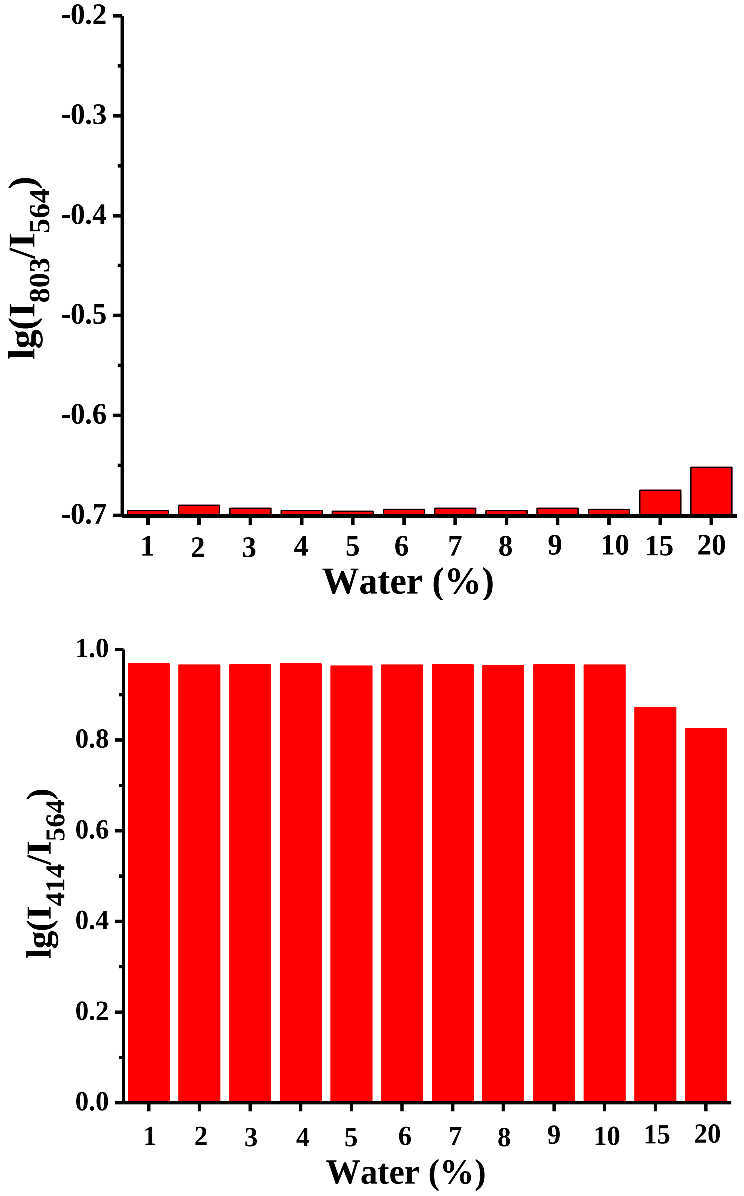

Fig. S5 The fluorescence spectra of **SY** (5 μM) to CN^- (10 μM) in acetonitrile with different percentage of water at 803 nm (I_{803}/I_{564}, top figure) and 564 nm (I_{414}/I_{564}, bottom figure).

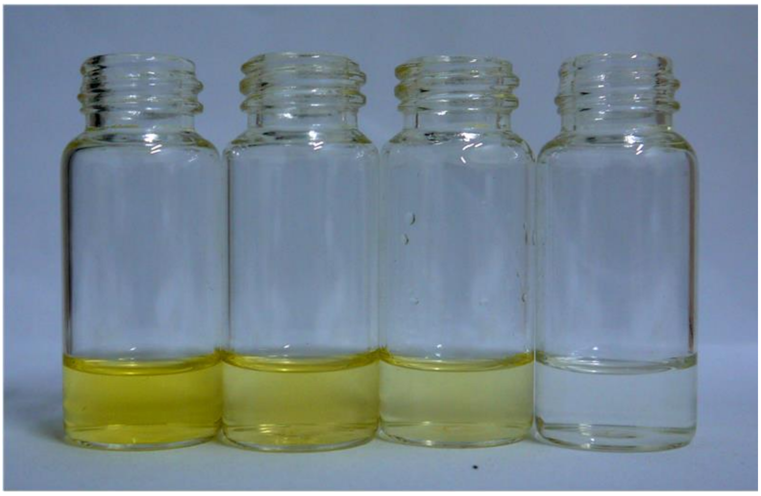

Fig. S6 The color changes under natural light of **SY** (5 μM) with the addition of 0−10 μM CN^- (from left to right).

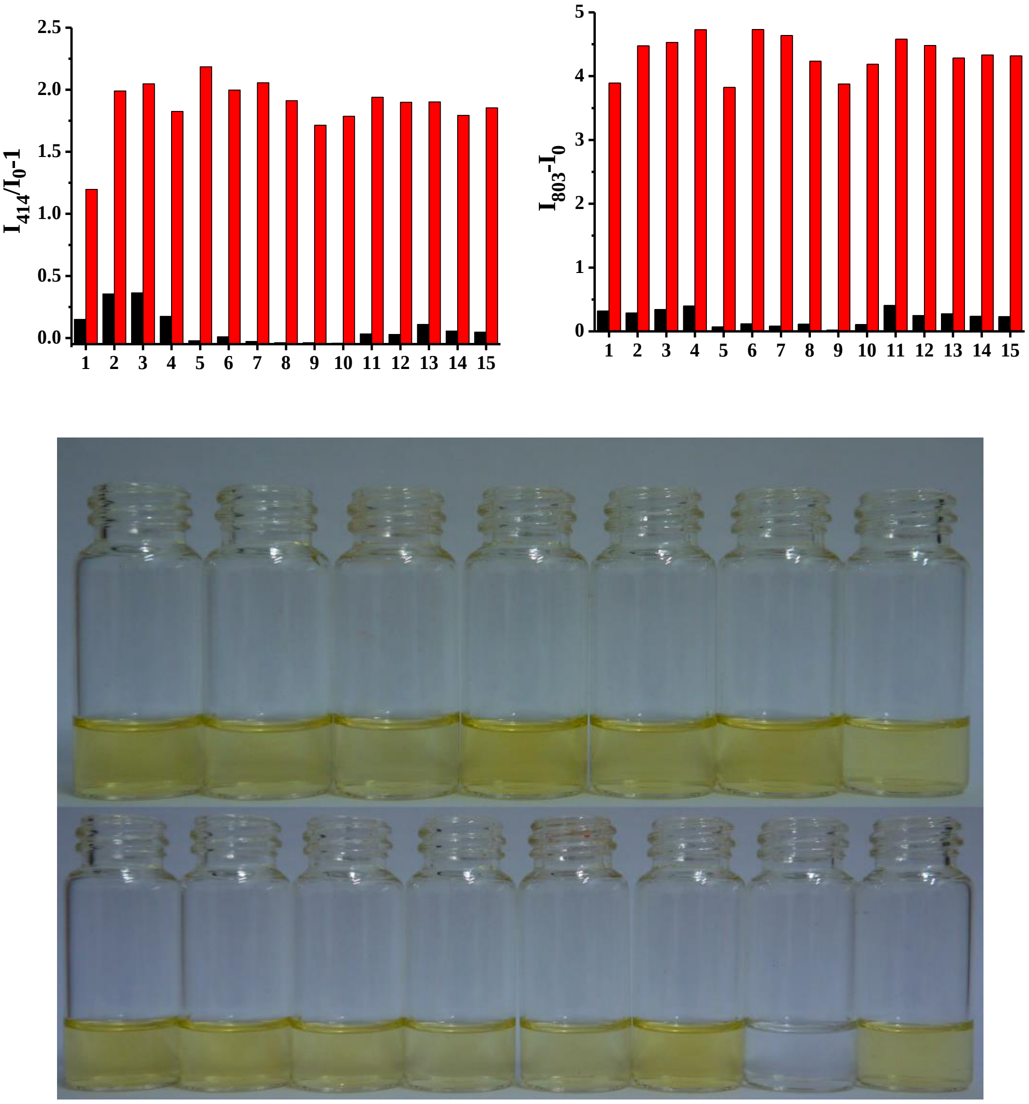

Fig. S7 The selectivity of probe **SY** (5 μM) for CN^-. Black bars represent the addition of a single analyte (25 μM for each), red bars represent the subsequent addition of CN^- (5 μM) to the mixture. All spectra were recorded instantaneously after the addition of anions in acetonitrile at room temperature. 1, S^{2-}; 2, CO_3^{2-}; 3, ACO^-; 4, $S_2O_3^{2-}$; 5, SO_4^{2-}; 6, I^-; 7, Br^-; 8, Cl^-; 9, $H_2PO_4^-$; 10, F^-; 11, SCN^-; 12, NO_2^-; 13, GSH; 14, Hcy; 15, Cys. λ_{ex}= 360 nm. The bottom figure is the color changes under natural light of **SY** (5 μM) with other interference ions (25 μM for each) and CN^- (5 μM).

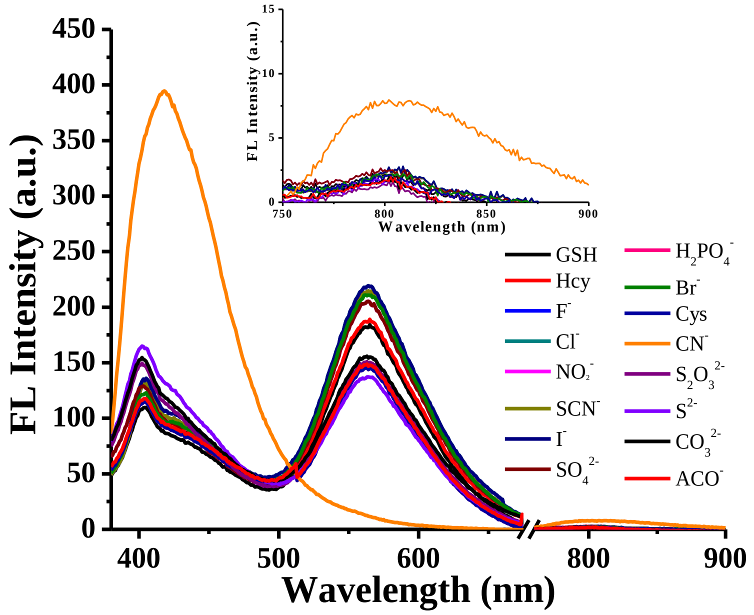

Fig. S8 The fluorescence spectra of probe **SY** (5 μM**)** for CN^{-} (10 μM) and other analytes (25 μM for each) in acetonitrile. Inset: enlarged fluorescence spectra from 750 nm to 900 nm, λ_{ex} = 360 nm.

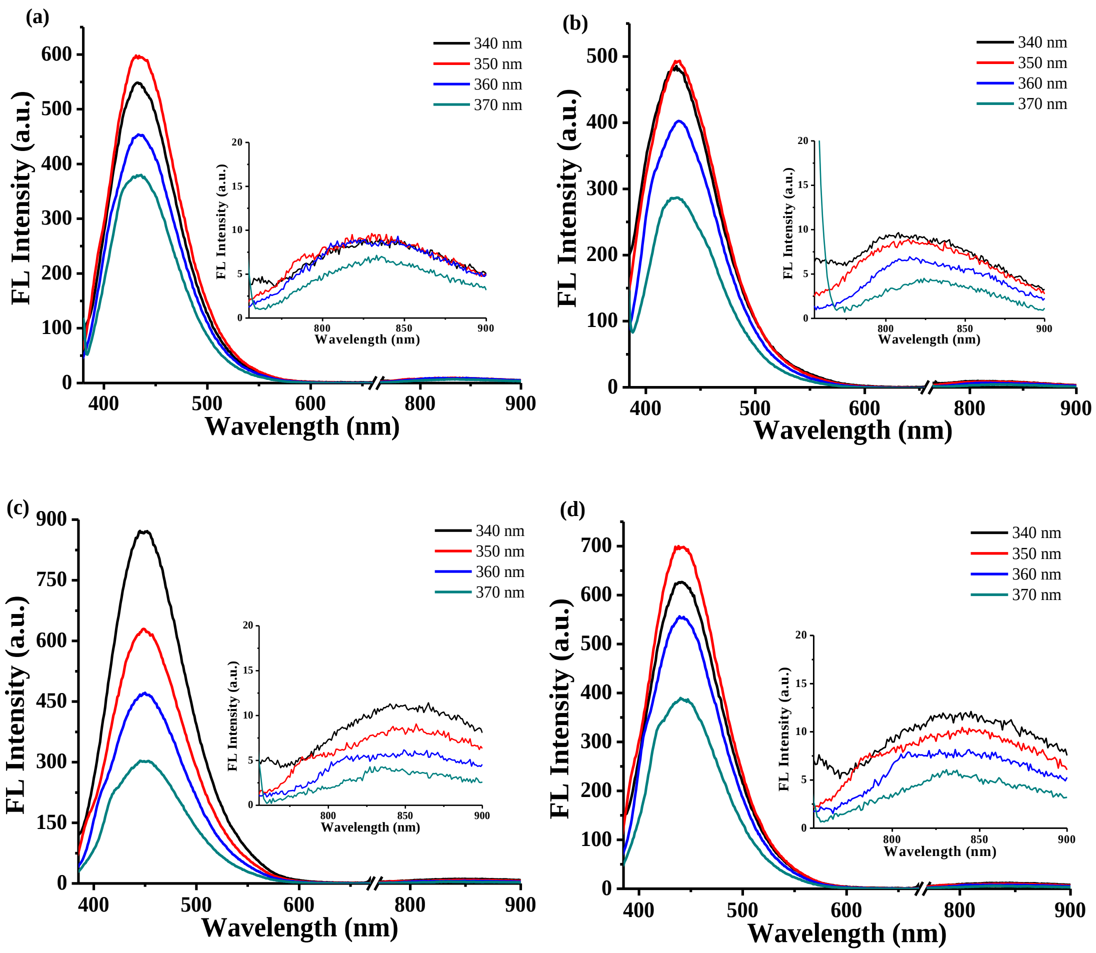

Fig. S9 Fluorescence spectra of **(A)** (2 μM) recorded under different excitation in different solvent. (a) acetonitrile; (b) dichloromethane; (c) methanol; (d) ethanol. Inset: enlarged fluorescence spectra from 750 nm to 900 nm, λ_{ex} = 360 nm.

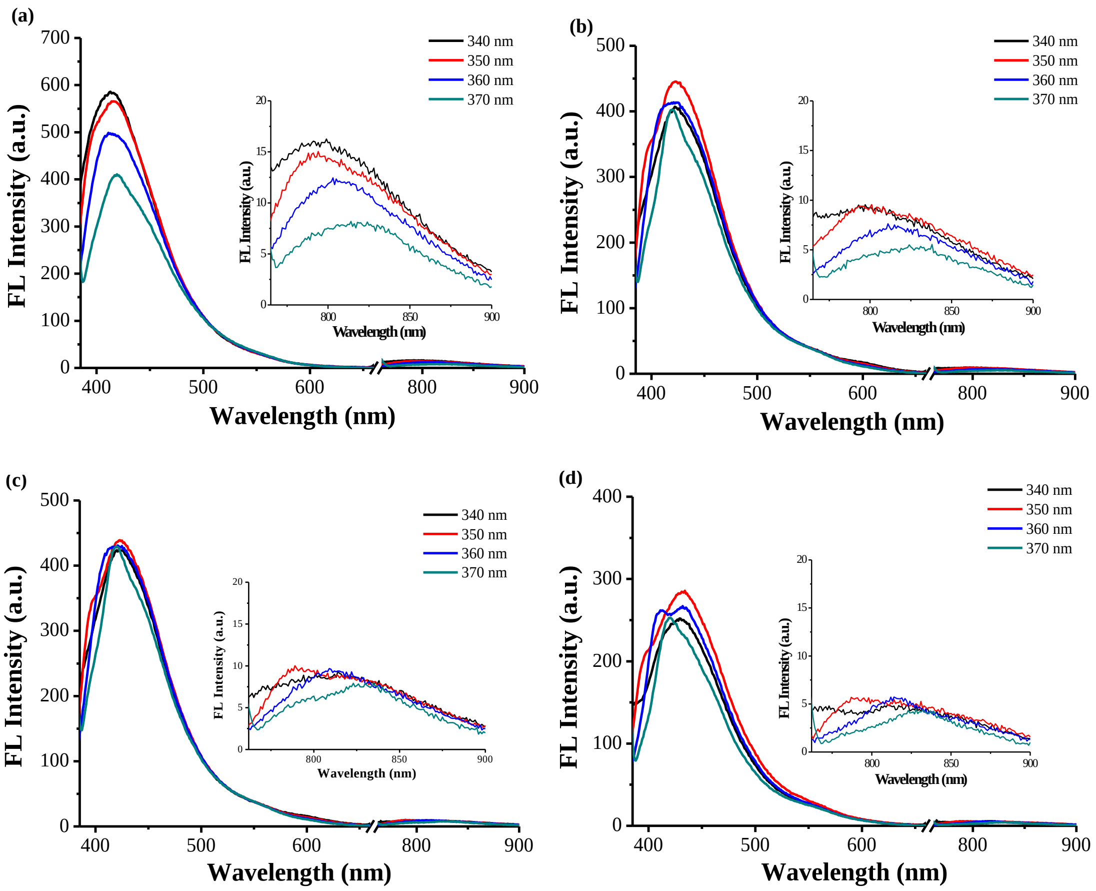

Fig. S10 Fluorescence spectra **SY** (5 μM) with the addition of CN^- (10 μM) recorded under different excitation in different solvent. (a) acetonitrile; (b) dichloromethane; (c) methanol; (d) ethanol. Inset: enlarged fluorescence spectra from 750 nm to 900 nm, λ_{ex} = 360 nm.

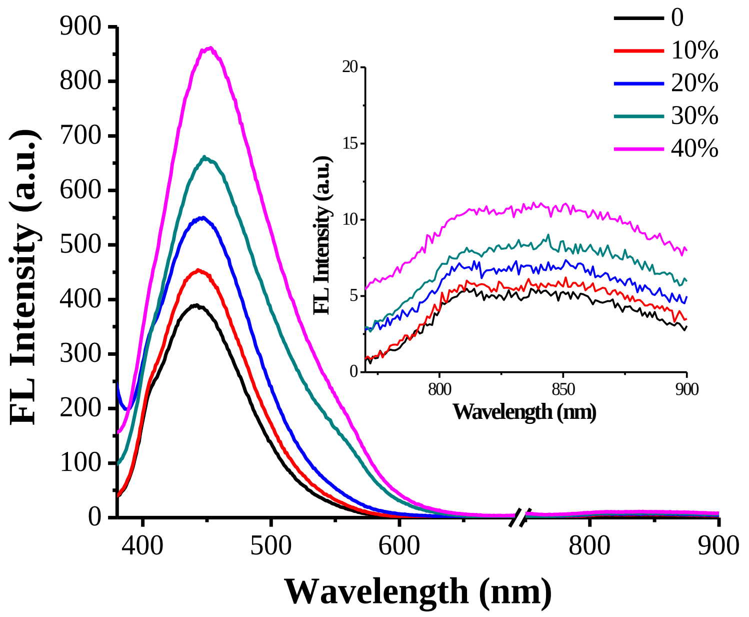

Fig. S11 Fluorescence spectra of **(A)** (2 μM) with different percentage of glycerol in ethanol-glycerol system. Inset: enlarged fluorescence spectra from 770 nm to 900 nm, λ_{ex} = 360 nm. Slits: 5/10 nm.

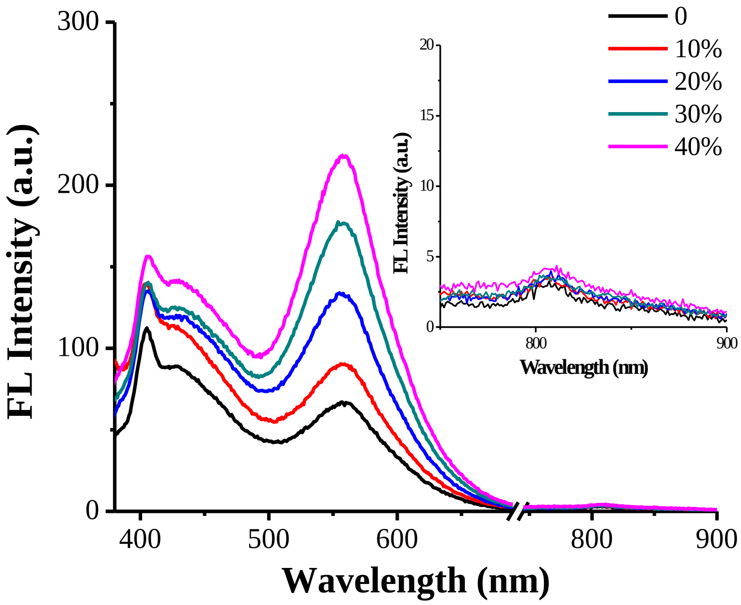

Fig. S12 Fluorescence spectra of **SY** (5 μM) with different percentage of glycerol in ethanol-glycerol system. Inset: enlarged fluorescence spectra from 750 nm to 900 nm, λ_{ex} = 360 nm. Slits: 5/10 nm.

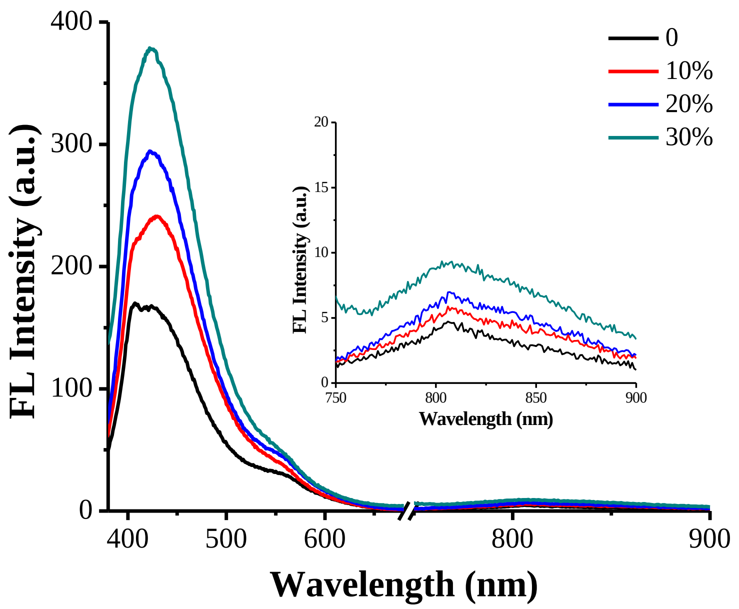

Fig. S13 Fluorescence spectra of **SY** (5 μM) + CN^{-} (10 μM) with different percentage of glycerol in ethanol-glycerol system. Inset: enlarged fluorescence spectra from 750 nm to 900 nm, λ_{ex} = 360 nm. Slits: 5/10 nm.

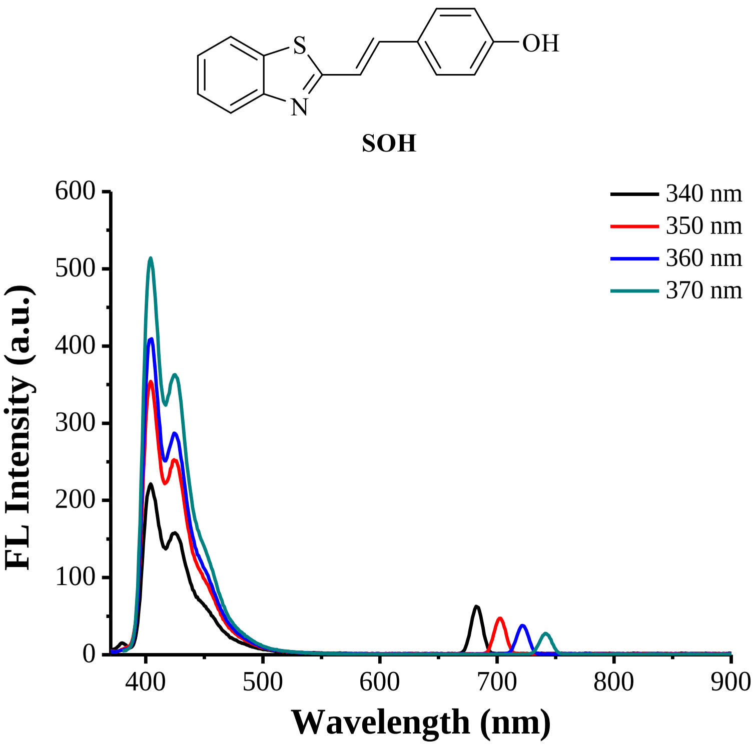

Fig. S14 Fluorescence spectra of **SOH** (2 μM) recorded under different excitation in acetonitrile.

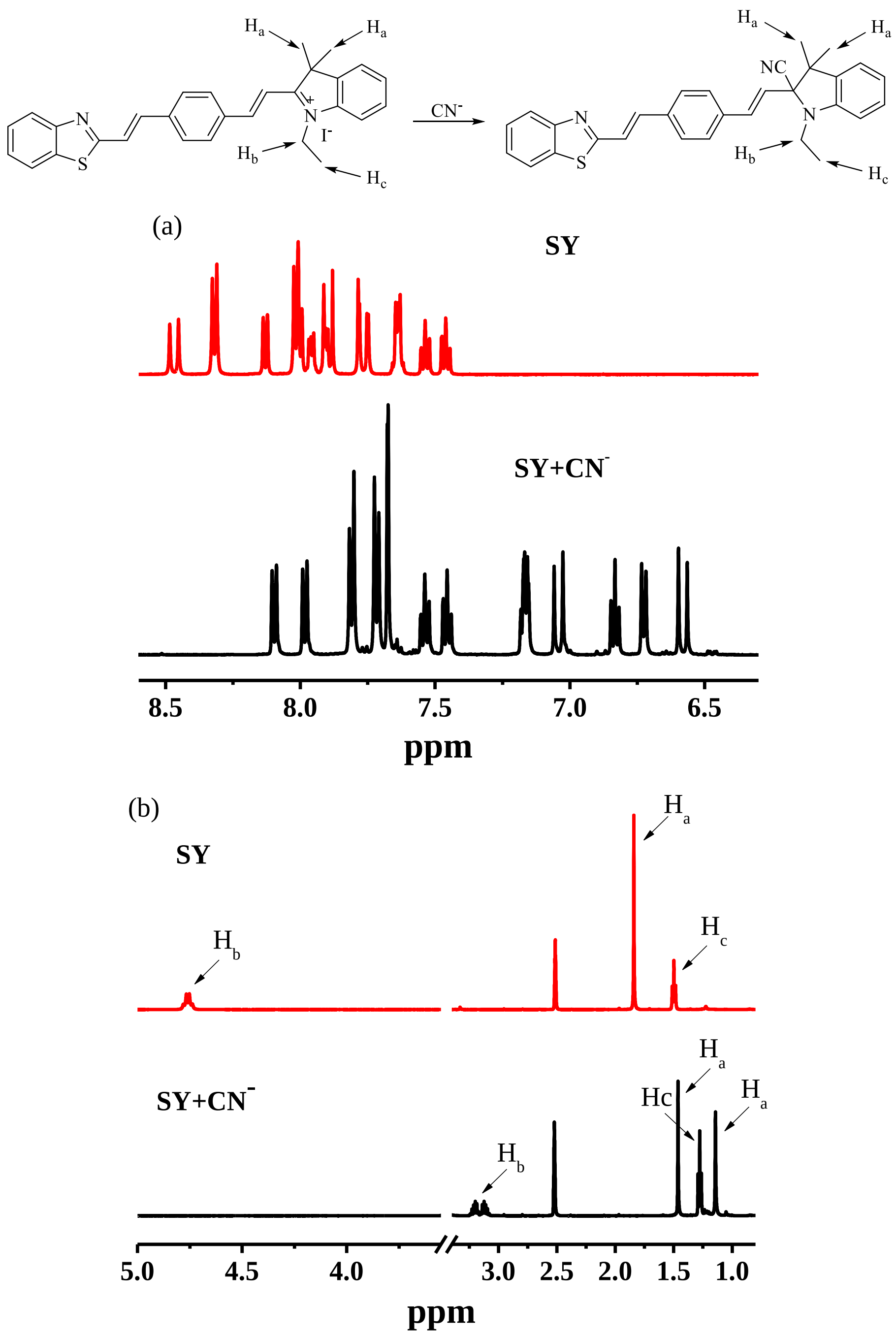

Fig. S15 ^{1}H-NMR spectra changes of **SY** and **SY** + CN^{-} in DMSO-d_6.

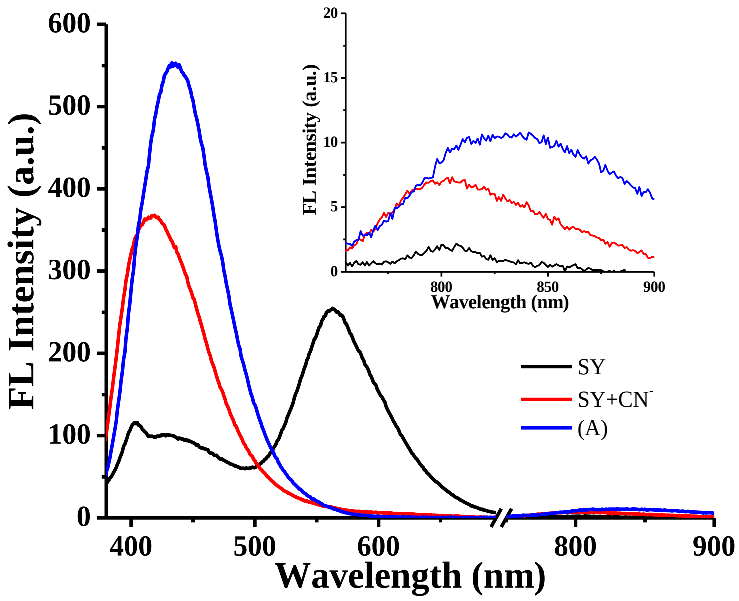

Fig. S16 Fluorescence spectra of **SY**, **SY** + CN^- and **(A)** in acetonitrile at room temperature. Black line represents the spectra of 5 μM **SY**, red line represents the spectra of 5 μM **SY** with the addition of 10 μM CN^-, blue line represents the spectra of 2 μM **(A)**. Inset: fluorescence spectra from 750 nm to 900 nm, λ_{ex} = 360 nm.

5. ^{1}H NMR, ^{13}C NMR and MS spectra

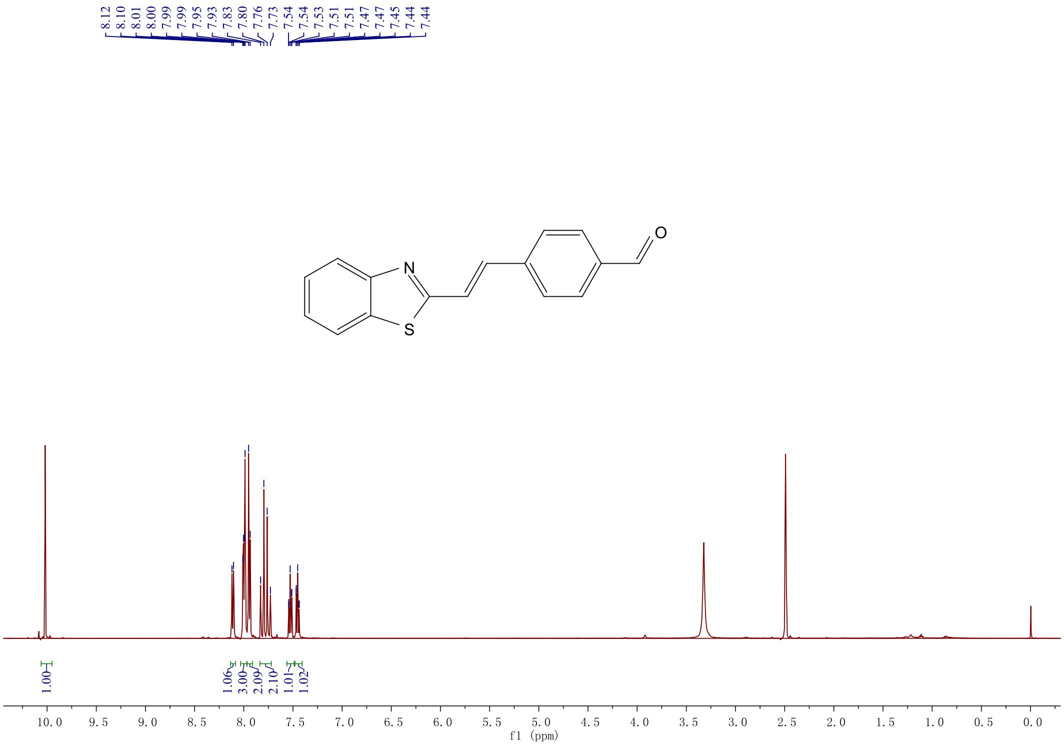

Fig. S17 ^{1}H NMR of (A) in DMSO-d_6.

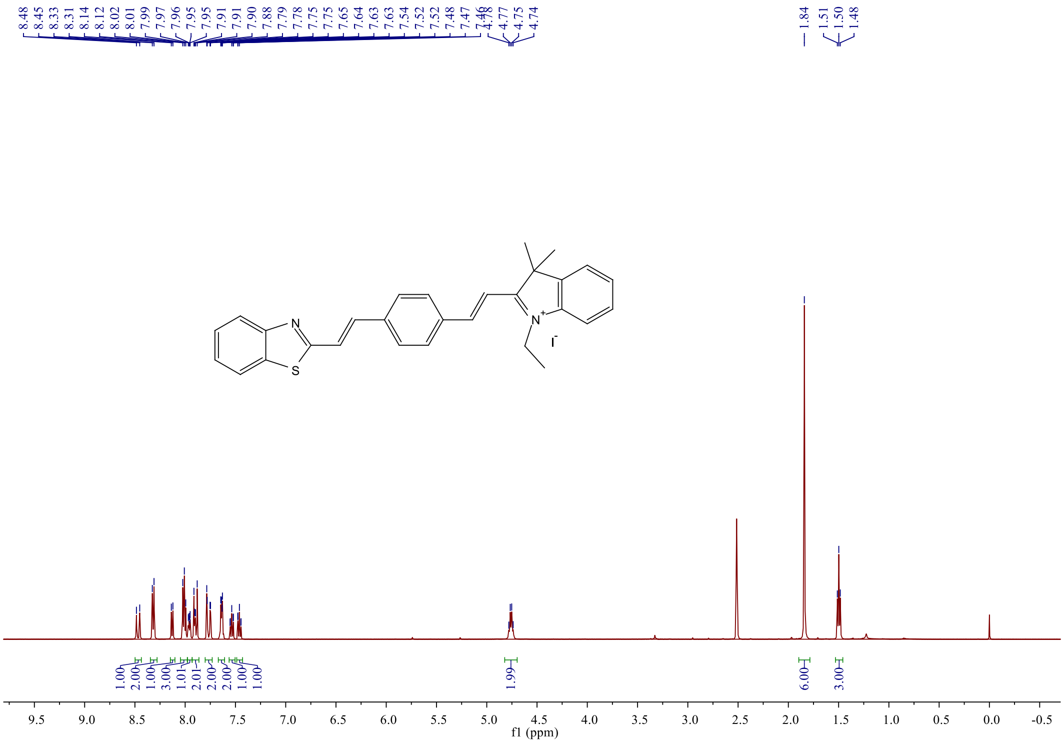

Fig. S18 ^{1}H NMR of **SY** in DMSO-d_6.

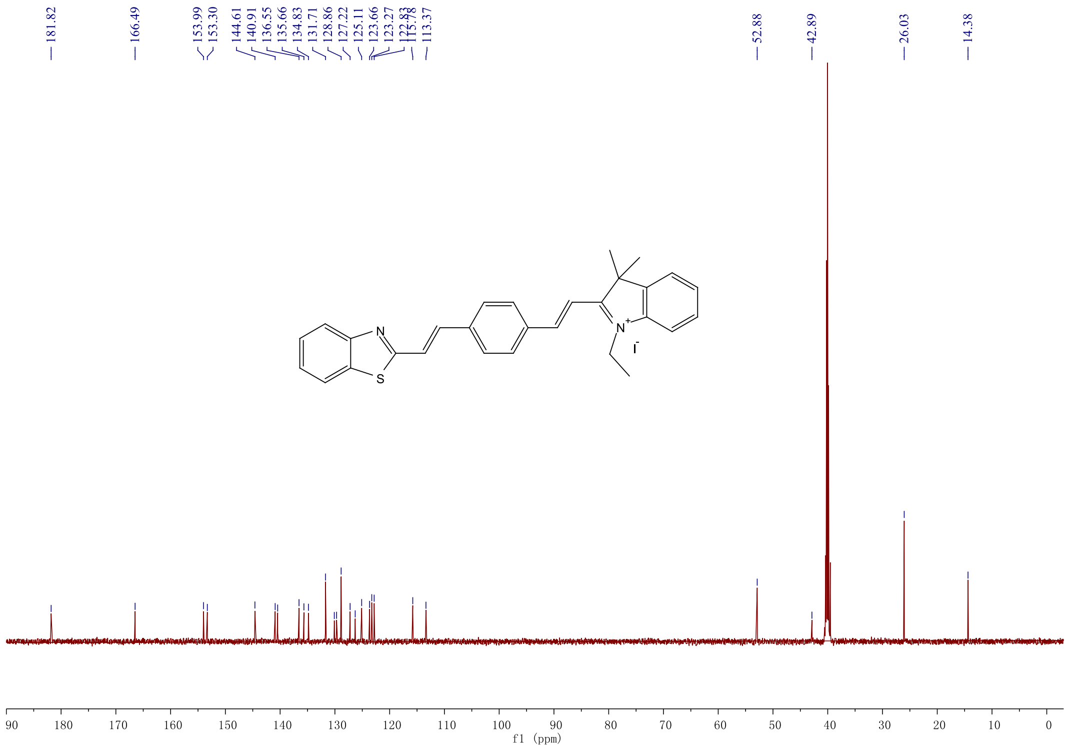

Fig. S19 ^{13}C NMR of **SY** in DMSO-d_6.

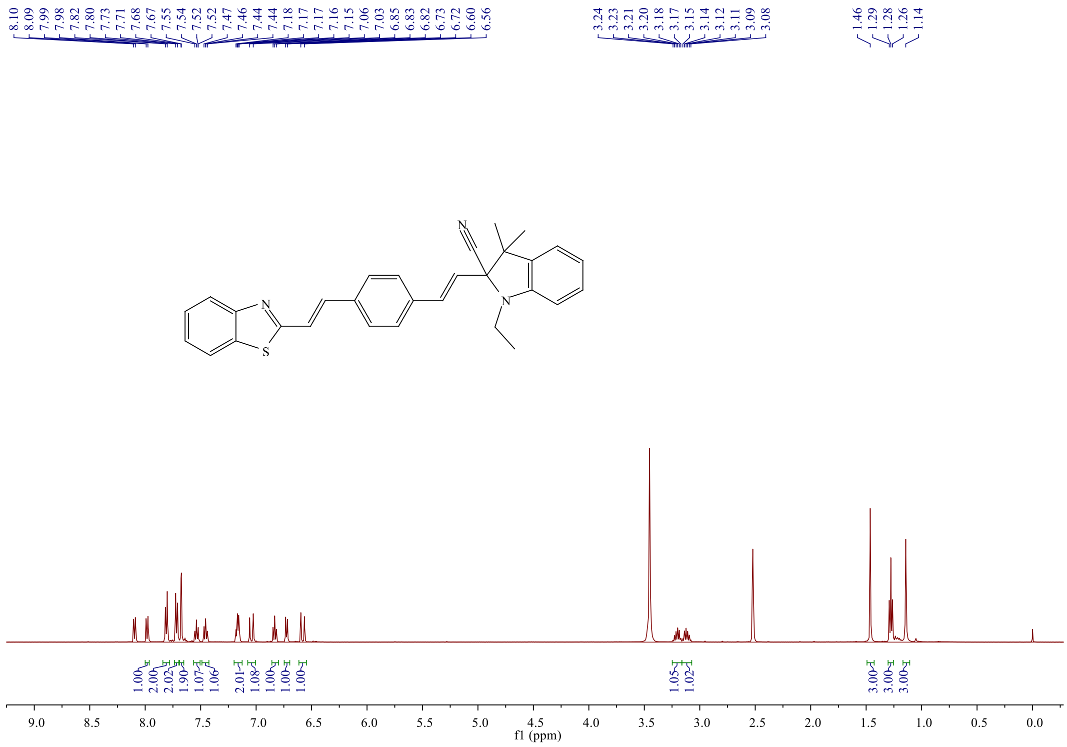

Fig. S20 ^{1}H NMR of **SY** in the presence of CN^{-} in DMSO-d_6.

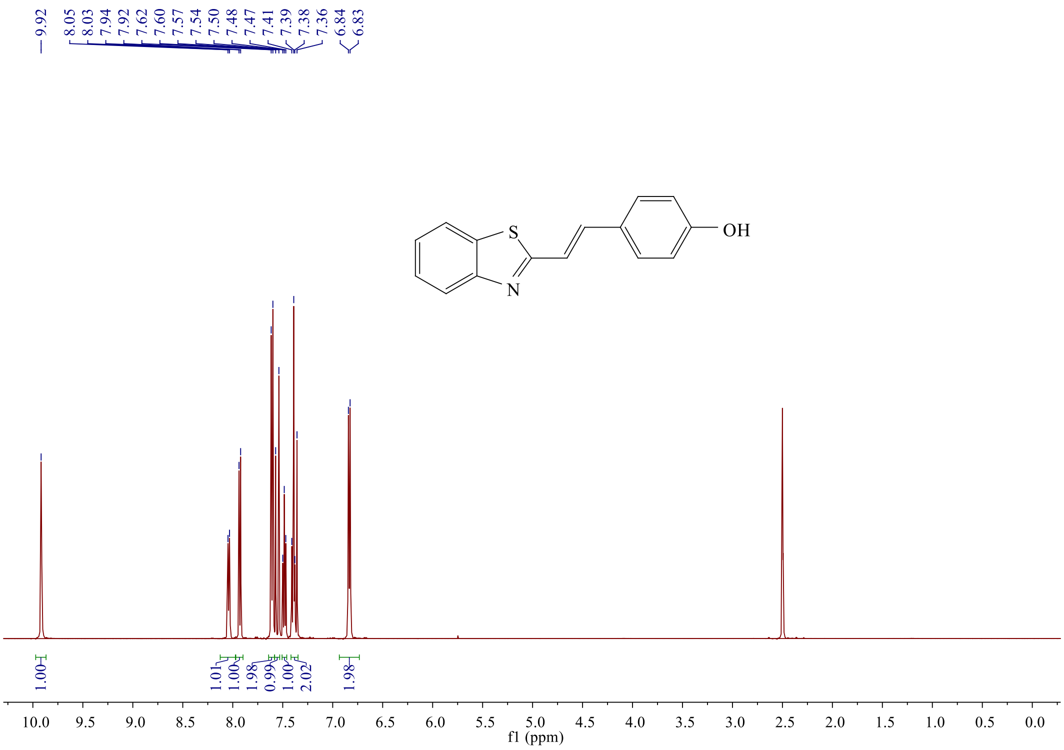

Fig. S21 ^{1}H NMR of **SOH** in DMSO-d_6.

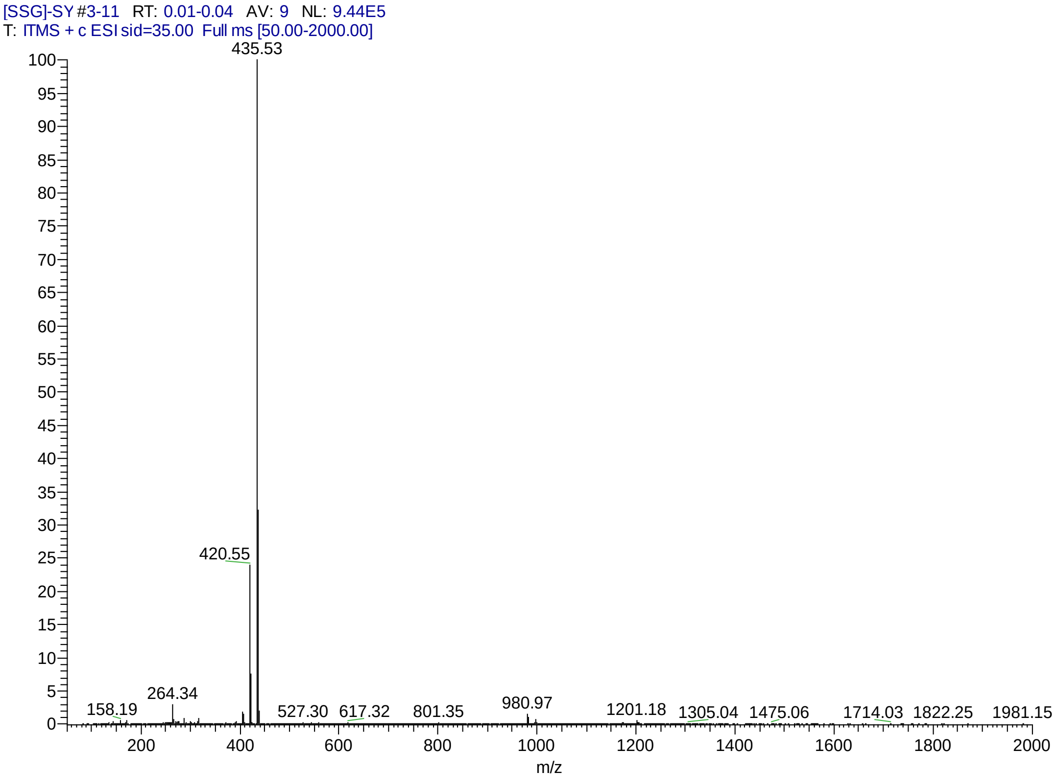

Fig. S22 ESI-MS of **SY**.

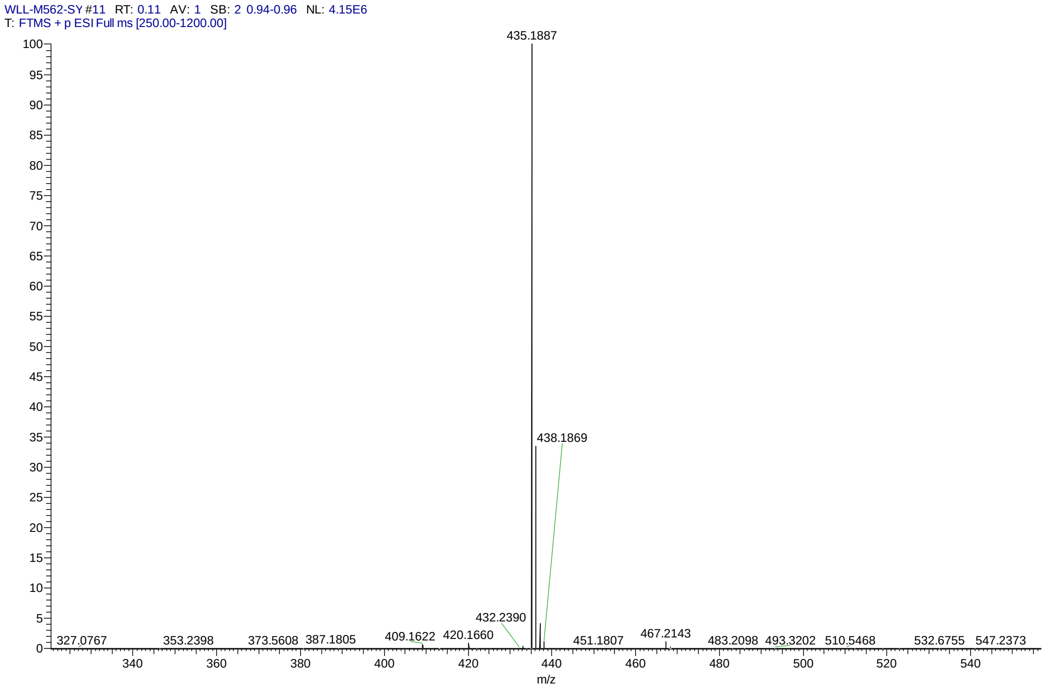

Fig. S23 HRMS of **SY**.

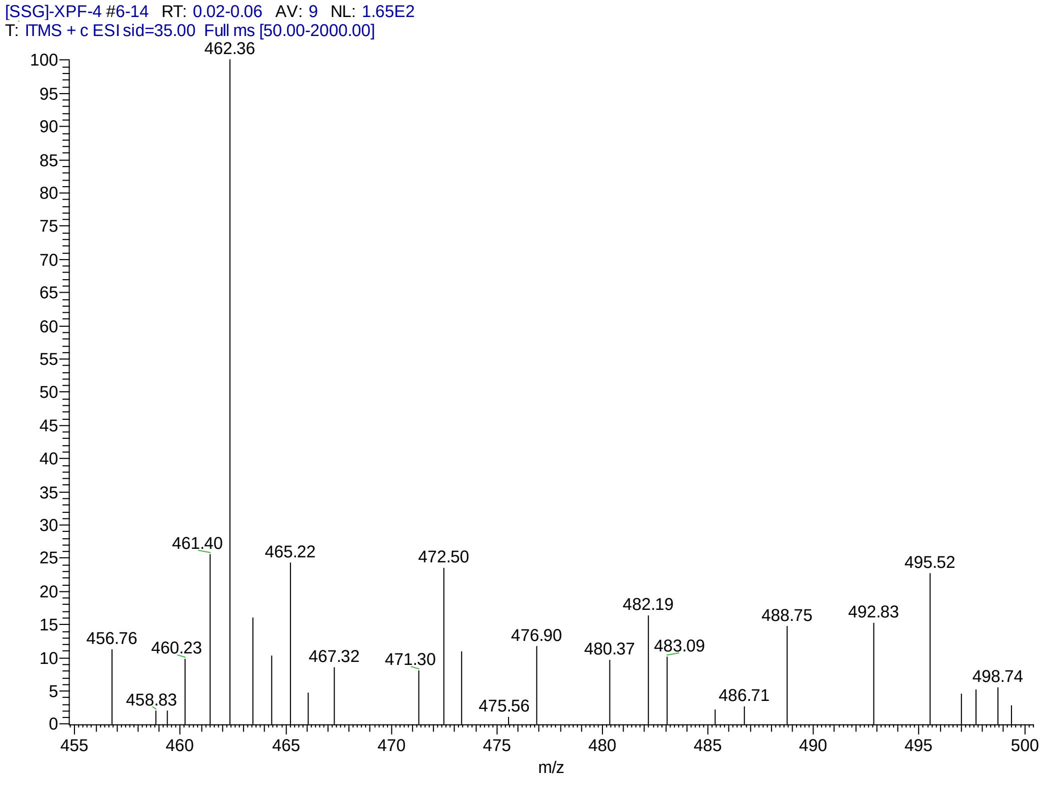

Fig. S24 ESI-MS of **SY** + CN^{-}.

Table S1 β-Glucosidase assays in commercial amygdalin samples.

samples	β-glucosidase (mU)	theoretical value (μM)	CN^- (I_{414}, μM)	RSD (I_{414}, %)	CN^- (I_{564}, μM)	RSD (I_{564}, %)	CN^- (I_{803}, μM)	RSD (I_{803}, %)
			1.1		1.0		1.1	
1	20	1.0	1.2	8.5	0.9	5.1	1.0	8.2
			1.2		1.0		0.9	
			5.4		5.2		5.7	
2	100	5.0	5.3	4.1	5.0	1.9	5.5	6.5
			5.5		5.1		5.8	
			6.9		7.7		8.2	
3	150	7.5	6.8	5.7	7.4	2.0	8.0	5.9
			7.6		7.7		7.2	

References

1. Zhang, L. *et al.* Synthesis, characterization and fluorescence adjustment of well-defined polymethacrylates with pendant π-conjugated benzothiazole via atom transfer radical polymerization (ATRP). *Polymer* **50**, 4807-4812 (2009).

2. Thomsen, V., Schatzlein, D. & Mercuro, D. Limits of detection in spectroscopy. *Spectroscopy* **18**, 112-114 (2003).

3. Hakonen, A. Plasmon enhancement and surface wave quenching for phase ratiometry in coextraction-based fluorosensors. *Anal. Chem.* **81**, 4555-4559 (2009).

4. Goswami, S. *et al.* CHEF induced highly selective and sensitive turn-on fluorogenic and colorimetric sensor for Fe^{3+}. *Dalton Trans.* **42**, 15113-15119 (2013).
